# Correlation between circulating cell‐free *PIK3CA* tumor DNA levels and treatment response in patients with *PIK3CA*‐mutated metastatic breast cancer

**DOI:** 10.1002/1878-0261.12305

**Published:** 2018-05-04

**Authors:** Annette R. Kodahl, Sidse Ehmsen, Niels Pallisgaard, Anne M. B. Jylling, Jeanette D. Jensen, Anne‐Vibeke Lænkholm, Ann S. Knoop, Henrik J. Ditzel

**Affiliations:** ^1^ Department of Oncology Odense University Hospital Denmark; ^2^ Department of Cancer and Inflammation Institute of Molecular Medicine University of Southern Denmark Odense Denmark; ^3^ Department of Clinical Biochemistry Køge Hospital Denmark; ^4^ Department of Pathology Odense University Hospital Denmark; ^5^ Department of Surgical Pathology Zealand University Hospital Slagelse Denmark; ^6^ Department of Oncology Rigshospitalet Copenhagen Denmark; ^7^ Academy of Geriatric Cancer Research (AgeCare) Odense University Hospital Denmark

**Keywords:** ddPCR, liquid biopsy, metastatic breast cancer, PIK3CA

## Abstract

Liquid biopsies focusing on the analysis of cell‐free circulating tumor DNA (ctDNA) may have important clinical implications for personalized medicine, including early detection of cancer, therapeutic guidance, and monitoring of recurrence. Mutations in the oncogene, *PIK3CA*, are frequently observed in breast cancer and have been suggested as a predictive biomarker for PI3K‐selective inhibitor treatment. In this study, we analyzed the presence of *PIK3CA* mutations in formalin‐fixed, paraffin‐embedded, metastatic tissue and corresponding ctDNA from serum of patients with advanced breast cancer using a highly sensitive, optimized droplet digital PCR (ddPCR) assay. We found 83% of patients with *PIK3CA* mutation in the metastatic tumor tissue also had detectable *PIK3CA* mutations in serum ctDNA. Patients lacking the *PIK3CA* mutation in corresponding serum ctDNA all had nonvisceral metastatic disease. Four patients with detectable *PIK3CA‐*mutated ctDNA were followed with an additional serum sample during oncological treatment. In all cases, changes in *PIK3CA* ctDNA level correlated with treatment response. Our results showed high concordance between detection of *PIK3CA* mutations in tumor tissue and in corresponding serum ctDNA and suggest that serum samples from patients with advanced breast cancer and ddPCR may be used for *PIK3CA* mutation status assessment to complement imaging techniques as an early marker of treatment response.

**Abbreviations**
bp: base pairs
CT: computed tomography
ctDNA: circulating tumor DNA
ddPCR: droplet digital polymerase chain reaction
FFPE: formalin‐fixed and paraffin‐embedded
HE: hematoxylin–eosin
HER2: human epidermal growth factor receptor 2
PET: positron emission tomography
PTEN: phosphatase and tensin homolog
SOP: standard operating procedure

## 1 Introduction

Cell‐free DNA is released from both normal and cancer cells into the circulation (Choi *et al*., 2005; Stroun *et al*., 2000). In contrast to benign tumors and other noncancerous conditions, a rapid turnover of tumor cells is thought to result in a consistently increased release of circulating tumor DNA (ctDNA; Diehl *et al*., 2005). This enables clinical use of repeated and noninvasive blood samples as a ‘liquid biopsy’ to monitor the dynamic evolution of human cancers (Siravegna *et al*., 2017) and response to treatment, which could accompany the well‐established RECIST 1.1 and PERCIST 1.0 criteria evaluative of changes in tumor burden during cancer treatment (Eisenhauer *et al*., 2009).

In some cancers, certain driver mutations are found at high frequencies, such as KRAS in colorectal cancer (Hao *et al*., 2017). In other cancers, including breast cancer, the pattern of driver mutations is more diverse. The most frequent driver mutation observed in breast cancer other than *TP53* is *PIK3CA,* an oncogene encoding the p110α component of the phosphoinositide 3‐kinase (PI3K). Genetic alterations in PI3K pathway constituents, for example, PI3K‐activating mutations, loss of the antagonistic tumor suppressor phosphatase, and tensin homolog or via the transduction of aberrant receptor tyrosine kinase signals, result in upregulation of the PI3K pathway (Saal *et al*., 2005; Stemke‐Hale *et al*., 2008). Abnormal activation of the PI3K pathway is a common finding in a variety of tumor types, including breast cancer, and the recent BELLE‐2 study of the PI3K inhibitor, Buparlisib, showed promising results in progression‐free survival in combination with endocrine therapy, especially in patients with tumors exhibiting *PIK3CA* mutations (Baselga *et al*., 2017). Additional PI3K inhibitors are currently under clinical development (Zhao *et al*., 2017).

*PIK3CA* mutations are present in approximately 30–40% of all breast cancers (Arsenic *et al*., 2014; Buttitta *et al*., 2006; Campbell *et al*., 2004; Jensen *et al*., 2011; Koboldt *et al*., 2012; Li *et al*., 2006; Maruyama *et al*., 2007; Saal *et al*., 2005), with the highest frequency in estrogen receptor (ER)‐positive and human epidermal growth factor receptor (HER)2‐positive breast cancers (Koboldt *et al*., 2012; Saal *et al*., 2005). The four most frequent ‘hotspot’ *PIK3CA* mutations are located within two exons (exon 9: E545K and E542K and exon 20: H1047R and H1047L) and account for 80–90% of all *PIK3CA* mutations in human malignancies (Kalinsky *et al*., 2009; Karakas *et al*., 2006). We (Kodahl *et al*., 2015) and others (Oshiro *et al*., 2015) have previously reported disputed points concerning the use of ctDNA containing *PIK3CA* mutations as a biomarker in early‐stage breast cancer patients. The ctDNA amount is influenced by the extent of the disease, including tumor burden, and generally, very little tumor DNA is present in the circulation in early‐stage breast cancers compared to advanced stage (Diehl *et al*., 2005). Increasing the blood sample volume to be analyzed as well as technological improvements that increase sensitivity to detection of ctDNA in low blood concentrations is necessary to achieve better assay results (Han *et al*., 2017). However, ctDNA containing *PIK3CA* mutations has been suggested to be a promising biomarker of cancer patients with advanced disease both for early detection of recurrence and to monitor treatment response (Board *et al*., 2010; Dawson *et al*., 2013; Higgins *et al*., 2012), and studies reveal a correlation of *PIK3CA* mutation level and tumor burden in advanced breast cancer (Garcia‐Saenz *et al*., 2017; Higgins *et al*., 2012).

This study aimed to investigate whether *PIK3CA* mutations identified in archived formalin‐fixed, paraffin‐embedded (FFPE), metastatic tissue samples of breast cancer patients could also be detected in their corresponding ctDNA from serum samples using an optimized droplet digital PCR (ddPCR) assay (Kodahl *et al*., 2015). Furthermore, we sought to investigate whether the level of *PIK3CA*‐mutated ctDNA could be used as an early marker for monitoring treatment response and supporting clinicians in guiding treatment decisions.

## 2 Materials and methods

### 2.1 Patient samples

Metastatic tumor biopsies and corresponding serum samples were obtained from 66 patients who were part of a prospective study, in which patients from 2007 to 2013 were offered a biopsy from the metastasis, when diagnosed with recurrent breast cancer at the Department of Oncology at Odense University Hospital. Sixteen of the included 66 patients had previously had their metastatic tumor tissue analyzed for *PIK3CA* mutations using pyrosequencing (Jensen *et al*., 2011). In this study, the tumor biopsies and corresponding serum samples from these 16 patients, in addition to the other 50 patients, were analyzed for *PIK3CA* mutations using ddPCR (Section 3.3).

Blood was drawn into BD Vacutainer™, SST™ Serum Separation Tubes with polymer gel/silica activator. According to standard operating procedure, serum was prepared within one hour of sample collection after centrifugation (2000G, 1800 ***g***; 10 min at 20 °C) and immediately stored at −80 °C.

Hematoxylin–eosin (HE) sections were prepared from FFPE, tissue biopsies from metastatic sites and reviewed by a skilled breast pathologist to confirm the presence and amount of tumor tissue. Cancer cell content in all cases was > 30%. For DNA extraction, 2 × 10 μm sections of FFPE samples from each tumor sample were used. Follow‐up data, including patient outcome, were available for all patients, and observations on follow‐up were censored at date of data withdrawal (March 2018). Overall survival is defined as time to death. An overall survival curve was generated by Kaplan–Meier survival analysis using stata statistical software (StataCorp LLC, College Station, TX, USA). The experiments were undertaken with the understanding and written consent of each patient. The study methodologies conformed to the standards set by the Declaration of Helsinki. The study was approved by The Regional Scientific Ethical Committee for Southern Denmark (ID: S‐20070003) and the Danish Data Protection Agency.

### 2.2 Droplet digital PCR of FFPE samples

Genomic DNA was extracted using a Maxwell 16 FFPE Tissue LEV DNA Purification Kit (Promega, Madison, WI, USA) according to the manufacturer's recommendations. Purified DNA was eluted in 100 μL and analyzed by ddPCR in a QX100 droplet digital system (Bio‐Rad, Copenhagen, Denmark) using PrimePCR™ ddPCR™ Mutation Assays (Bio‐Rad) for wild‐type *PIK3CA* as well as the four most common mutations in the *PIK3CA* gene: E542K (c.1624G>A) Assay ID PIK3CA:dHsaCP2000073 (mut) and dHsaCP2000074 (wt), chromosome location 3:178936050–178936172, amplicon length 80 bp, E545K (c.1633G>A) Assay ID PIK3CA:dHsaCP2000075 (mut) and dHsaCP2000076 (wt), chromosome location 3:178936068–178936190, amplicon length 80 bp, H1047L (c.3140A>T) Assay ID PIK3CA:dHsaCP2000123 (mut) and dHsaCP2000124 (wt), chromosome location 3:178952024‐178952146, amplicon length 74 bp and H1047R (c.3140A>G) Assay ID PIK3CA:dHsaCP2000077 (mut) and dHsaCP2000078 (wt), chromosome location 3:178952065‐178952187, amplicon length 80 bp. They were all performed in duplicates, as described by the manufacturer. Five microlitre sample DNA was combined with 1 μL 20× wild‐type primers/probe mix (HEX‐labeled) and 1 μL 20× target primers/probe mix (FAM‐labeled) along with 10 μL 2× ddPCR supermix for probes (Bio‐Rad) and 3 μL DNase‐/RNase‐free sterile water in a 20 μL reaction volume. Droplets were generated from the 20 μL reaction mix and 70 μL droplet generator oil for probes (Bio‐Rad) in a droplet generator DG8 cartridge (Bio‐Rad), and the droplets were transferred into a microtiter plate and subjected to thermal cycling, as recommended by the manufacturer. Thermal cycle parameters were as follows: enzyme activation 95 °C for 10 min, followed by 40 cycles of denaturation 94 °C for 30 s and annealing/extension 55 °C for 60 s with a ramp rate of 2 °C/s, and a final enzyme inactivation step 98 °C for 10 min. After PCR thermocycling, the emulsions were enumerated by fluorescence measurement using a QX100 (Bio‐Rad) droplet reader. Mutant populations were identified, and the fractional abundance calculation of mutant to total *PIK3CA* molecules was calculated for each sample using Poisson statistics from the qx100 software (Bio‐Rad).

### 2.3 Droplet digital PCR of serum samples

**Figure 1:**
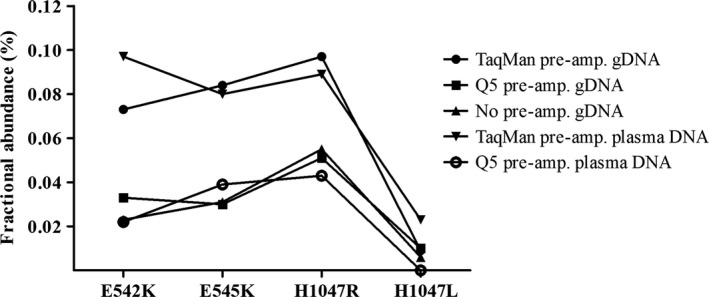
Assay specificity for *PIK3CA* mutations. Comparison of mutant *PIK3CA* detection in human control genomic DNA (gDNA) and cell‐free plasma DNA following pre‐amplification or no pre‐amplification using Q5 High‐Fidelity DNA Polymerase (New England BioLabs) or TaqMan^®^ PreAmp Master Mix (Life Technologies). The level of blank (LoB) was 0.04% for the mutation E545K and for H1047R, 0.02% for E542K, and less than 0.01% for H1047L as shown by the graphs.

DNA was purified from 1 mL of serum using a MagNA Pure LC Total Nucleic Acid Isolation Kit – large volume (Roche) according to the manufacturer's instructions. Water was added to serum samples with inadequate volumes to 1 mL prior to purification. Also prior to purification, 5 μL non‐human exogenous internal control DNA spike‐in fragment of 191 bp was added to each serum sample. The control fragment was part of the soya bean CPP1 gene and generated by PCR (Pallisgaard *et al*., 2015). Purified DNA was eluted in 100 μL in the supplied elution buffer. Twenty‐five microlitre of the purified DNA was used to control for loss during purification by measuring the amount of the added spike‐in fragment by qPCR, contamination of lymphocyte DNA by qPCR analysis of the immunoglobulin gene rearrangements of B cells and number of genomic DNA alleles per ml of serum, as previously described (Pallisgaard *et al*., 2015). To increase the sensitivity of the analysis, the remaining 75 μL serum DNA was subjected to 12 cycles of PCR pre‐amplification with Q5 High‐Fidelity DNA Polymerase (New England BioLabs, Ipswich, MA, USA) using a multiplex *PIK3CA* primer mix. The PIK3CA ddPCR assay has been extensively validated as reported in Kodahl *et al*. (2015). In short, serum DNA was up‐concentrated by centrifugation 10 min 12 000 ***g*** using a Amicon Ultra‐0.5 Centrifugal Filter Unit (Merck Millipore, Burlington, MA, USA) and volume was adjusted to 20 μL with DNase‐/RNase‐free sterile water. A multiplex pre‐amplification step was incorporated prior to ddPCR using the *PIK3CA* E542K and H1047L PrimePCR™ ddPCR™ Mutation Assays (Bio‐Rad) primers, diluted 1 : 9. Pre‐amplification was performed in 50 μL reactions using 20 μL of up‐concentrated serum DNA, 5 μL multiplex primer mix (100 nm final concentration of each primer), and 25 μL Q5^®^ Hot Start High‐Fidelity 2X Master Mix (New England BioLabs). Thermal cycling was performed by enzyme activation 98 °C for 10 min, followed by 12 cycles of denaturation 98 °C for 15 s, annealing 55 °C for 60 s and extension 72 °C for 60 s, and a final enzyme inactivation step 99.9 °C for 10 min. The Q5 High‐Fidelity DNA Polymerase (New England BioLabs) and TaqMan^®^ PreAmp Master Mix (Life Technologies, Carlsbad, CA, USA) were tested for potential incorporation of technical mutations due to misincorporation of the Taq polymerase during the pre‐amplification step by comparing the mutant allele fraction in samples from healthy donors before and after pre‐amplification (Fig. 1). It should be noted that as the linearity of the pre‐amplification step was not experimentally verified there may be a slight risk of a quantification bias due to nonperfect linear pre‐amplification. However, as the wild‐type and mutant amplicon only differs by one nucleotide in the middle of the amplicon, it is very unlikely that the mutant/wild‐type ratio will be affected during the pre‐amplification step. The pre‐amplified products were then diluted 50‐fold, and ddPCR was performed in duplicates for *PIK3CA* E542K, E545K, H1047L, and H1047R mutation detection, according to the manufacturer's protocol (Bio‐Rad). The results were reported as a fractional abundance of mutant DNA alleles to total (mutant plus wild‐type) DNA alleles. Positive control DNA fragments for the four PIK3CA mutations were constructed and spiked into DNA from healthy donors and used as positive controls, as previously described (Spindler *et al*., 2012).

From the nonmutated alleles, the level of blank (LoB) for each of the four *PIK3CA* mutation assays was determined using pre‐amplified cell‐free plasma DNA and the Q5 High‐Fidelity DNA Polymerase (New England BioLabs). The LoB % for each sample was calculated by the formula: ((mut allele)/((mut allele) + (wt allele))) × 100. The standard deviation of the average LoB % was calculated, and the level of detection (LoD, with 95% confidence) was determined by (LoB + (2 × Std.dev of LoB) (Table S1). LoD of the ddPCR assay allowed detection of a mutant allele fraction of ≥ 0.08% (Table S1) corresponding to one mutant molecule in a background of 1200 wild‐type molecules.

### 2.4 Controls

Four 180‐bp DNA fragments containing one of the four *PIK3CA* mutations analyzed in this study were generated by site‐directed PCR mutagenesis, as previously described (Spindler *et al*., 2012). The mutated PCR fragments were spiked into normal donor DNA and used as positive controls throughout the study. In all ddPCR and qPCR, positive and negative (water) and wild‐type (normal donor DNA) controls were performed in parallel.

## 3 Results

### 3.1 Patient characteristics

**Figure 2:**
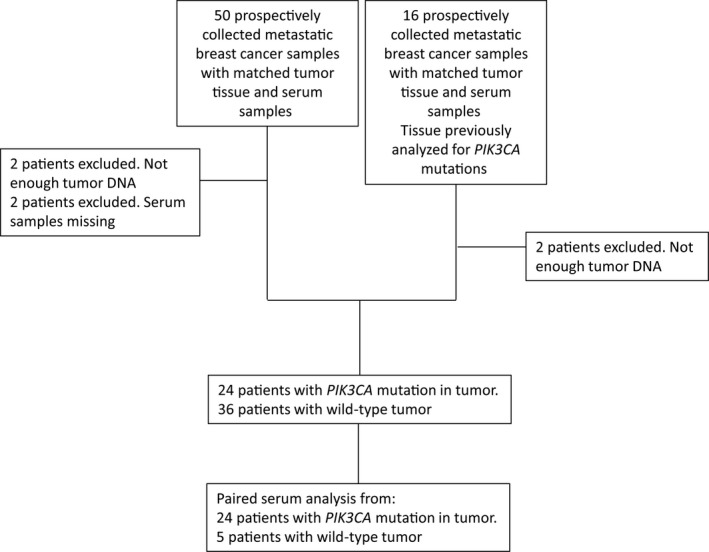
Consort diagram. A total of 66 metastatic tumor biopsies and corresponding serum samples were prospectively collected. Of these, 24 metastasis with *PIK3CA* mutation and five wild‐type as determined by ddPCR were selected for further analysis.

Archived formalin‐fixed, paraffin‐embedded metastatic tumor biopsies and corresponding serum samples were available from 66 patients with metastatic disease who were part of a prospective study (2007–2013) at the Department of Oncology, Odense University Hospital, Denmark (Fig. 2). Metastatic tissue samples from 16 patients had, as part of a previous study (Jensen *et al*., 2011), been tested for the presence of *PIK3CA* mutations by pyrosequencing, 15 of which were found to be *PIK3CA*‐mutation positive and one wild‐type. In this study, 50 additional patients were included to a total of 66 patients. Of these 66 patients, four samples were excluded due to less than 10% detectable tumor cells in the FFPE tumor tissue, and two patients were excluded due to missing serum samples, resulting in a total of 60 matched samples. Serum samples from 24 patients with a *PIK3CA* mutation in their metastatic tissue and five patients with *PIK3CA* wild‐type metastatic tissue (randomly selected) were analyzed for circulating cell‐free *PIK3CA* mutations using ddPCR (Fig. 2). The survival curve from Kaplan–Meier estimates of the 29 metastatic breast cancer patients included for further analysis showed a median overall survival of 20 months (Fig. S1).

### 3.2 *PIK3CA* mutation analysis of metastatic breast cancer samples

*PIK3CA* mutations were observed in 40% of the total metastatic tissue samples (24/60), with 33% (8/24) having exon 9 mutations (E542K and E545K) and 67% (16/24) having exon 20 mutations (H1047L and H1047R), as determined by the optimized ddPCR method. However, the inclusion of the preselected samples might account for the high overall observed frequency of *PIK3CA* mutations in tumor tissue compared to the unselected samples (24%; 11/46).

Of the 24 breast cancer patients with *PIK3CA* mutation‐positive metastasis, 92% were ER‐positive (*n* = 22), 8% were ER‐negative (*n* = 2), and 8% were HER2‐amplified (*n* = 2) (Table 1). The first blood sample from each patient was taken an average of 1.5 days after diagnostic biopsy (range: 26 days before to 43 days after diagnosis) (Table 2). Of the 24 patients with detectable *PIK3CA* mutations in the metastatic tumor tissue, 20 (83%) had similar mutations detectable in corresponding ctDNA from serum. Four patients with *PIK3CA* mutations in metastatic tumor tissue had no detectable mutations in their corresponding serum samples, and interestingly, all had nonvisceral metastatic disease. No patients with *PIK3CA* wild‐type tumor biopsies (*n* = 5) exhibited *PIK3CA* mutations in their corresponding serum samples.

**Table 1:** Clinical characteristics of breast cancer patients with metastatic tissue and corresponding ctDNA from serum analyzed for *PIK3CA* mutation

Patient no.	ER status	HER2 status	Site of metastatic disease	Site of biopsy
1	+	+	Non‐visceral	Lymph nodes
2	+	−	Non‐visceral	Bone
3	+	−	Non‐visceral	Bone
4	+	−	Non‐visceral	Lymph nodes
5	−	−	Non‐visceral	Subcutis, chest wall
6	+	−	Visceral, Non‐visceral	Liver
7	+	−	Non‐visceral	Bone
8	+	−	Visceral	Pleural effusion
9	−	−	Visceral, Non‐visceral	Lymph nodes
10	+	−	Non‐visceral	Bone
11	+	NA	Visceral, Non‐visceral	Lymph nodes
12	+	−	Visceral, Non‐visceral	Bone
13	+	−	Visceral, Non‐visceral	Liver
14	+	−	Non‐visceral	Bone
15	+	−	Non‐visceral	Bone
16	+	−	Visceral, Non‐visceral	Liver
17	+	−	Visceral, Non‐visceral	Liver
18	+	−	Non‐visceral	Subcutis, chest wall
19	+	−	Visceral, Non‐visceral	Liver
20	+	−	Non‐visceral	Lymph nodes
21	+	−	Visceral, Non‐visceral	Bone
22	+	−	Visceral, Non‐visceral	Liver
23	+	−	Visceral, Non‐visceral	Liver
24	+	+	Non‐visceral	Skin
25	+	−	Non‐visceral	Bone
26	−	−	Visceral, Non‐visceral	Liver
27	+	−	Visceral, Non‐visceral	Bone
28	−	−	Visceral, Non‐visceral	Subcutis
29	+	−	Visceral, Non‐visceral	Lymph nodes

ER+ cutoff was ≥1% positive tumor cells. Visceral metastases are metastases in the lung, liver, brain and/or peritoneum. Non‐visceral localizations are the skin, lymph nodes, soft tissue and/or bone. NA, not available.

**Table 2:** *PIK3CA* mutational status and treatment response of breast cancer patients with metastatic tissue and corresponding ctDNA from serum analyzed for *PIK3CA* mutation

Patient no.	Tumor tissuemutation	Serum ctDNAmutation	Previousregisteredmutation	Serum 1(days)	Serum 1ctDNA(copies/μl)	Serum 2(days)	Serum 2ctDNA(copies/μl)	Serum 3(days)	Serum 3ctDNA(copies/μl)	Treatmenttype	Treatment start (days)	Status(days)	Imagingtype	Response
1	E542K	E542K		−4	1.6									
2	E545K	E545K		−6	49									
3	H1047R	H1047R		0	13.6									
4	WT	No	H1047R	−3										
5	E542K	No		−14										
6	E542K	E542K/ E545K	E542K	−2	21/230									
7	H1047L	H1047L		−2	761									
8	WT	No	E545K	18										
9	WT	No		−10										
10	H1047R	H1047R	H1047R	0	3.1									
11	H1047L	H1047L		36	5									
12	E545K	E545K	E545K/ H1047R	−12	125.1									
13	H1047R	H1047R		−2	1170									
14	E542K	No		43										
15	H1047R	No		−1										
16	H1047R	H1047R		−5	17.4									
17	H1047R	H1047R		−1	9500									
18	H1047R	No		−13										
19	E545K	E545K		−3	694									
20	H1047R	H1047R		−10	1.8									
21	H1047L	H1047L		−3	21.8									
22	E545K	E545K		−14	114.2									
23	H1047R	H1047R	H1047R	−2	15.4									
24	H1047R	H1047R		15	26.5	36	3.9			Docetaxel	15	76	CT	PR
25	H1047R	H1047R		−26	22.5	2	3			AI	−26	96	CT	PR
26	H1047R	H1047R		0	566	90	2.8			Epirubicin, Cyclophosphamide	12	74	PET	PR
27	H1047R	H1047R		−22	343	33	41.3	47	9.2	Paclitaxel	13	67	PET	PR
28	WT	No		16		53				Capecitabine	33	83	CT	PD
29	WT	No		16		127				AI	8	86	CT	NC

Day 0 refers to the day of diagnostic biopsy from a metastatic lesion. Serum samples and treatment start are given in days after biopsy. No serum ctDNA mutation refers to none measurable *PIK3CA* mutation in serum. Medical imaging (computed tomography (CT) or positron emission tomography (PET)) was used to measure the response and assessed according to RECIST 1.1 and PERCIST 1.0 criteria: PR, partial response; PD, progressive disease; NC, no change; AI, aromatase inhibitor; WT, wild‐type.

### 3.3 *PIK3CA*‐mutated ctDNA as an early marker for treatment response

**Figure 3:**
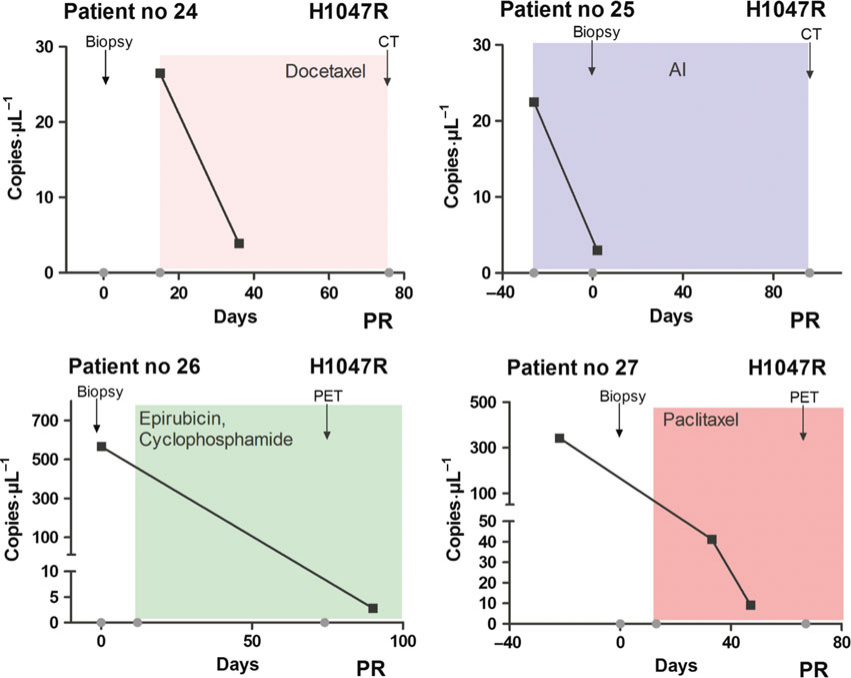
Serial monitoring of *PIK3CA* point mutation levels and correlation with treatment response in patients with advanced breast cancer. Measurements of serial fractional abundance of *PIK3CA‐*mutated ctDNA (H1047R) and evaluation of treatment response by CT or PET imaging in four patients (patient nos 24, 25, 26, and 27). Blood samples were collected two to three times per patient and the level of *PIK3CA‐*mutated ctDNA in serum reported as copies·μL^−1^ (■). Details regarding type of treatment (docetaxel, aromatase inhibitor (AI), epirubicin, cyclophosphamide, or paclitaxel) and treatment schedule are indicated by colored shading. Time for biopsy and CT or PET imaging is indicated by arrows. All patients showed partial response (PR) after treatment.

Six patients had serial serum samples collected during oncological treatment (four patients with detectable ctDNA containing *PIK3CA* mutations and two wild‐type that remained wild‐type) (Table 2). Response evaluation was performed using computed tomography (CT) or positron emission tomography (PET) imaging according to RECIST 1.1 and PERCIST 1.0 (Eisenhauer *et al*., 2009) an average of 11.5 weeks after diagnostic biopsy (range: 9.5–15 weeks) (Table 2). Clinicopathological patient characteristics is provided in Table 1 and the results of the response evaluations is provided in Table 2. Correlation between ctDNA *PIK3CA* mutation level, treatment, and disease history for the four patients is shown in Fig. 3. Serum samples from all four patients (patient nos 24, 25, 26, and 27) showed a decreased level of *PIK3CA‐*mutated ctDNA in the second and, for one, third serum sample, suggesting tumor shrinkage during treatment. This was verified by CT or PET imaging showing partial response in all four patients during treatment within this follow‐up period (Table 2 and Fig. 3). Unfortunately, no additional serum samples were collected from these patients.

## 4 Discussion

Tissue biopsy is an invasive procedure that may cause distress as well as complications for the patient. Moreover, due to intratumoral heterogeneity, the use of tumor tissue to obtain an accurate genomic landscape of breast cancer could be challenging (Gerlinger *et al*., 2012). Accurate determination of the genomic landscape of breast tumors is important to identify driver mutations that may make them susceptible to targeted antitumor agents, but also to determine whether subclones within the metastasis subsequently acquire additional mutations that render the lesion drug‐resistant, leading to disease progression (Diaz *et al*., 2012). Metastatic lesions are, unlike primary tumors, generally not surgically accessible and must be treated with systemic therapies. To make decisions regarding targeted cancer treatment, liquid biopsies, including detection of mutant tumor DNA in the circulation, may be the future method of choice to determine optimal treatment strategy, monitor treatment response, and characterize escaping resistant subclones that may cause metastases.

In this study, we show that *PIK3CA* mutations can be identified in ctDNA of serum samples from patients with *PIK3CA*‐mutated metastatic breast cancer and that the circulating *PIK3CA* mutation level might be used to follow treatment response in these patients. Using an optimized, highly sensitive, ddPCR assay, we found that 83% of patients with *PIK3CA* mutation in the metastatic tumor tissue had detectable circulating *PIK3CA* mutations in their corresponding serum sample, which underlines that the method is highly useful to obtain information regarding tumor characteristics and might be favored over the invasive and time consuming solid biopsies. Moreover, our study implies that ctDNA is a resource for therapeutic guidance in patients who may benefit from PI3K‐selective inhibitor treatment (Baselga *et al*., 2017).

Response evaluation during treatment of metastatic breast cancer is currently assessed primarily by visualization of imaging data and/or clinical examination of the patient at monthly intervals. Liquid biopsies of ctDNA may provide an additional method to monitor tumor response to treatment. Although only four patients with *PIK3CA* mutation in metastatic tumor tissue were followed with serial serum samples, all showed changes in *PIK3CA* ctDNA levels that correlated with treatment response according to imaging assessments. Similar results have been reported by others (Garcia‐Saenz *et al*., 2017), although no correlation between *PIK3CA* mutation levels and treatment response was observed in two of eight advanced breast cancer patients (Garcia‐Saenz *et al*., 2017). Cases with discordance between *PIK3CA* mutation level and treatment response could be explained by the technology used (Kodahl *et al*., 2015) or the disease stage (early‐stage versus advanced breast cancer; Garcia‐Saenz *et al*., 2017; Kodahl *et al*., 2015), or may reflect tumor evolution and heterogeneity within advanced breast cancer disease. However, liquid biopsies have previously been suggested to reflect the global (primary and metastatic sites) molecular status of cancer in terms of tumor heterogeneity better than a solid tumor biopsy (Crowley *et al*., 2013). While the four patients followed with serial serum samples all had H1047R mutations, we would expect similar results for patients with other hotspot *PIK3CA* mutations. Our results from analysis of the serial serum samples suggest that the level of circulating PIK3CA mutations could be a marker of disease burden in patients with PIK3CA mutant tumors. However, having a PIK3CA mutation does not necessarily mean that the only effective treatment is PI3K‐targeted and, in the case of the patients we studied, tumor reduction was caused by chemo or antihormonal therapies.

Similar to our study, good concordance between *PIK3CA* mutation status in formalin‐fixed, paraffin‐embedded, biopsies and ctDNA of patients with metastatic breast cancer has been observed by others (Garcia‐Saenz *et al*., 2017; Higgins *et al*., 2012). Interestingly, the patients with *PIK3CA* mutation identified in the metastatic tissue but in whom similar mutations could not be detected in corresponding serum ctDNA all had nonvisceral metastatic disease, suggesting that the location of the metastasis, in addition to size, may influence the amount of tumor DNA shed into the circulation. On the other hand, the sensitivity of the ddPCR assay allowed detection of a mutant allele fraction of > 0.084%, corresponding to one mutant molecule in a background of 1200 wild‐type molecules, making it highly sensitive. A higher concordance between *PIK3CA* mutation status in formalin‐fixed, paraffin‐embedded, biopsies and ctDNA might have been achieved if we had used plasma instead of serum, as serum DNA can be contaminated by blood cell DNA (Jen *et al*., 2000). However, the risk of contamination was limited as serum was isolated within one hour from blood drawing and subsequently immediately stored at −80 °C. Further, all DNA samples were tested for potential blood cell DNA contamination by a novel qPCR assay utilizing the immunoglobulin gene rearrangements in B cells (Pallisgaard *et al*., 2015).

## 5 Conclusions

In conclusion, our results show high concordance between tumor tissue and ctDNA mutation status and suggest that serum samples from advanced breast cancer patients and ddPCR may be used for *PIK3CA* mutation status assessment to complement imaging techniques as a tool for monitoring treatment response. However, as not all metastatic breast cancers are *PIK3CA‐*positive, additional biomarkers in the metastatic setting are needed.

## Conflict of interest

The authors declare no conflict of interest.

## Author contributions

ARK, SE, ASK, and HJD participated in the study design. ARK and SE coordinated the project, performed data interpretation, and wrote the first draft of the manuscript. NP performed molecular analysis. AMBJ, JDJ, and AVL provided samples and clinical and paraclinical information. NP and HD assisted in writing the manuscript, and all authors have read and approved the final manuscript.

## Supporting information


**Fig. S1.** Overall survival analysis of patients analyzed for circulating cell‐free *PIK3CA* mutations in serum.
**Table S1.** Level of detection and level of blank of the ddPCR assay.
