# Supplementary material for: Correlation between circulating cell‐free PIK3CA tumor DNA levels and treatment response in patients with PIK3CA‐mutated metastatic breast cancer
Source: Mol Oncol. 2018 May 4;12(6):925–35. doi: 10.1002/1878-0261.12305 (PMC5983134; doi:10.1002/1878-0261.12305)
Supplement: Supplementary file 1 — Fig. S1. Overall survival analysis of patients analyzed for circulating cell‐free PIK3CA mutations in serum. Table S1. Level of detection and level of blank of the ddPCR assay. [file MOL2-12-925-s001.pdf]

SUPPLEMENTARY INFORMATION

Kodahl AR *et al.* Correlation between circulating cell-free *PIK3CA* tumor DNA levels and treatment response in patients with *PIK3CA*-mutated metastatic breast cancer.

Table S1. Level of detection and level of blank of the ddPCR assay. From non-mutated serum samples the level of detection (LoD) was calculated based on the level of blanks (LoB) from pre-amplified cell-free plasma DNA using the Q5 High-Fidelity DNA Polymerase (New England BioLabs).

	E542K	E545K	H1047R	H1047L
**LoB (%)**	0.024	0.024	0.039	0.005
**Std.dev. (%)**	0.0097	0.0136	0.0188	0.0046
**LoD (%) (95% confidence)**	0.0435	0.0513	0.0763	0.0141
**n**	40	34	26	33

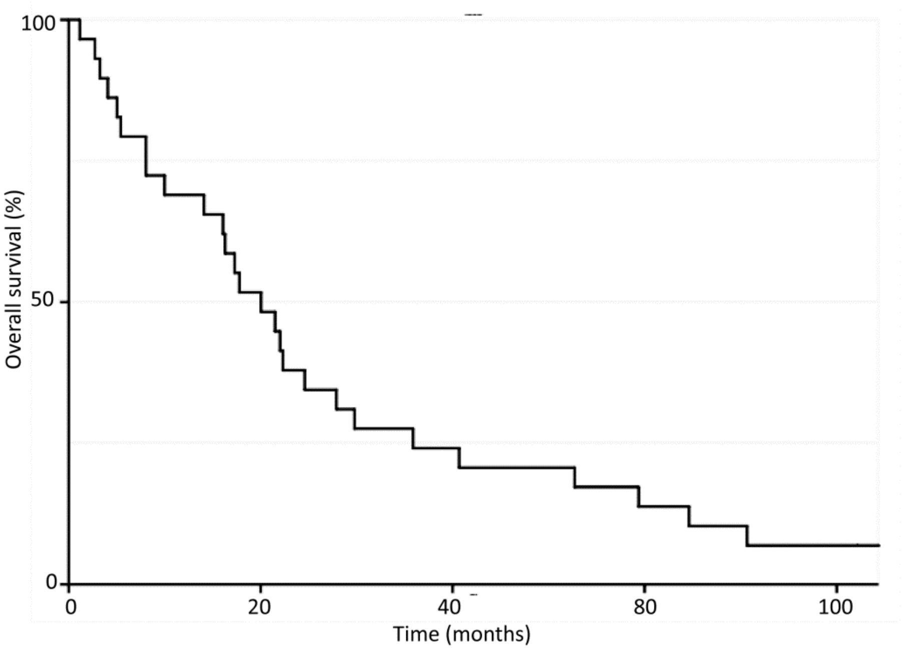

Figure S1. Overall survival analysis of patients analyzed for circulating cell-free *PIK3CA* mutations in serum. Kaplan-Meier curves illustrates overall survival of metastatic breast cancer patients with *PIK3CA* mutation and wild type (n=29). Median overall survival for all patients were 20 months.
